# Retraction: Prediction of thermal distribution and fluid flow in the domain with multi-solid structures using Cubic-Interpolated Pseudo-Particle model

**DOI:** 10.1371/journal.pone.0350714

**Published:** 2026-06-02

**Authors:** The PLOS One Editors

The *PLOS One* Editors retract this article [1] due to concerns about compromised peer review.

MB, MR, and SS did not agree with the retraction. QN and ATN either did not respond directly or could not be reached.
